# Scaling the *Moments That Matter^®^* early childhood development model: how communities’ monitoring for change contributes to sustainable impact

**DOI:** 10.3389/fpubh.2023.1165991

**Published:** 2023-05-12

**Authors:** Dawn E. Murdock, Kelvin Munsongo, George Nyamor

**Affiliations:** ^1^Episcopal Relief & Development, New York, NY, United States; ^2^Zambia Anglican Council Outreach Programmes, Lusaka, Zambia; ^3^Anglican Church of Kenya Development Services Nyanza, Kisumu, Kenya

**Keywords:** Early childhood Development, Scalable models, Faith-based approach, Community-led monitoring, measurement for change, parenting intervention, civil society-government coordination, community ownership

## Abstract

This paper presents a community case study of how the *Moments That Matter^®^* (MTM) Program community-led monitoring, evaluation and learning (MEL) system contributes to a scalable model with quality and sustainable impact. With a faith-based approach, MTM is an early childhood development program partnership of Episcopal Relief & Development which is rooted in parenting empowerment and community ownership. MTM empowers Primary Caregivers, strengthening nurturing care of some 60,000 children aged under three since 2012. Launched in Zambia, MTM has expanded to five other countries. Based on MTM Zambia and Kenya, this paper examines how an innovative, community-led MEL system functions to drive sustainable impacts and scaling. Measurement for change has been critical to the community MEL system. MTM is *people-centered* with community leaders, early childhood development service providers, volunteers and Primary Caregivers all setting their specific goals. The program is *inclusive* with all stakeholders engaged in monitoring and making adjustments; *interactive* with relationship-based social and behavior change strategies; *informative* with continuous data gathering used for decisions and problem-solving; and dynamic with built-in flexibility and an adaptation process. The community-led MEL propels scaling up through two channels: (1) *New communities for MTM program start up:* As MTM communities graduate to community ownership, program staff and budget are then invested in new marginalized and underserved rural areas. (2) *Deepening reach within MTM communities:* Over the first two cycles, communities transition to community ownership, then continue independently of staff and budget. They identify a new set of vulnerable Primary Caregivers of children under three and carry out the caregiver parenting support and learning activities. The success of the program’s community-led MEL in achieving sustainable change and fueling the program scale up hinges on three factors: (1) *Initiating the community-led MEL dimension at project start, gradually increasing the community role* while reducing the staff role. (2) *Provision of Community MEL capacity-building and effective, user-friendly* tools to be tailored locally. (3) *Three program stakeholder types leading MEL and collaborating closely with each other:* ECD Committees with MTM-trained faith leaders, ECD Promoters, and Primary Caregivers of children under three.

## 1. Introduction

The vast scope of early childhood development (ECD) challenges in sub-Saharan Africa is well-documented (1). With so many young children at risk, there is a tremendous need for strengthening nurturing care parenting. Yet, there is also vast untapped potential in faith-based and other community leadership and volunteerism. The *Moments That Matter^®^* Early Childhood Development Program Partnership (MTM) of Episcopal Relief and Development galvanizes rural communities and the most vulnerable families around their shared goal of young children thriving. MTM prioritizes responsive care, early learning and child safety and security in the home from birth to age three (2). Episcopal Relief and Development and its faith-based implementing partner organizations have co-designed and developed MTM through monitoring, evaluation, learning and adaptation loops. Over the last 10 years, MTM has expanded from Zambia to five other African countries, impacting more than 59,000 children under 3 years old with their Primary Caregivers (3). MTM is currently implemented by Episcopal Relief and Development’s in-country faith-based partner organizations in Ghana, Kenya, Malawi, Mozambique, Namibia and Zambia.

The MTM Program model is a community-led approach that transitions to community ownership over three implementation cycles. As such, MTM partner organizations and local communities have been co-developing an innovative community-led monitoring, evaluation and learning (MEL) process. This work is ongoing, which is strengthening the five Measurement for Change aspirations of being people-centered, inclusive, interactive, informative and dynamic (4). The community-led MEL functions at three levels: MTM community and faith leaders; MTM frontline volunteer implementers known as ECD Promoters; and Primary Caregivers^1^. ECD Promoters are a new type of volunteer, dedicated to parenting responsive care, early learning and child safety and security. They also refer caregivers to health and nutrition services as needed through community health workers and health clinics.

*Moments That Matter^®^* is currently implemented in Kenya by the Anglican Church of Kenya Development Services Nyanza (ADS Nyanza) and in Zambia by the Zambia Anglican Council Outreach Programmes (ZACOP). In 2021, the African Population and Health Research Center (APHRC) concluded an independent implementation research study of the MTM Programs in Kenya and Zambia, with a summary report available online (3, 5). Key findings include the following:

- The MTM Program was effective in achieving Primary Caregiver ECD parenting outcomes.
- MTM demonstrated significant achievements in catalyzing social and behavior change, at both community and family levels, by producing a community-owned nurturing care ecosystem in civil society and effectively linking with government ECD services.
- The program demonstrated to be acceptable, appropriate, feasible and sustainable.
- The model has scaled effectively by combining a required core set of strategies and quality standards with local adaptations.
- A critical driver of successful impact and scaling is the community-led structure of the program, which encourages ownership by communities and their own monitoring, evaluation and learning of a shared ECD vision.

Based on learnings from the APHRC study and internal assessments, Episcopal Relief and Development and its MTM partners have been improving the program and strengthening the community-led MEL system with measurement for change (M4C) dimensions. In line with the M4C informative aspiration, program staff with community volunteers and participants are drawing on multiple types of evidence gathering, such as externally conducted research and participatory community MEL. This case study presents key learnings from the community-led MEL of the previous MTM phase 2018–2021, with efforts underway since 2022, to strengthen the MEL process and deepen its measurement for change dimension.

The paper describes how community-led MEL processes reinforce both the motivation of caregivers and MTM volunteers to strengthen nurturing care, as well as their sense of ownership for sustaining and expanding the work. It illustrates the contributions of community-led MEL processes both to sustainable nurturing care social and behavior change and to successful scaling with impact through two channels: (a) expanding MTM to new communities and (b) engaging more vulnerable families within existing MTM communities.

## 2. *Moments That Matter^®^* Program context

*Moments That Matter^®^* is designed for rural communities and the key rural socio-demographics for MTM are similar across different African countries: smallholder farming, severe poverty, poor health and nutrition and HIV prevalence. Typically, health facilities are remote and community health workers have large caseloads. Religious institutions are often the only entity with a local permanent presence and their faith leaders are influential in their communities.

### 2.1. *Moments That Matter^®^* program model and theory of change

**Figure 1:**
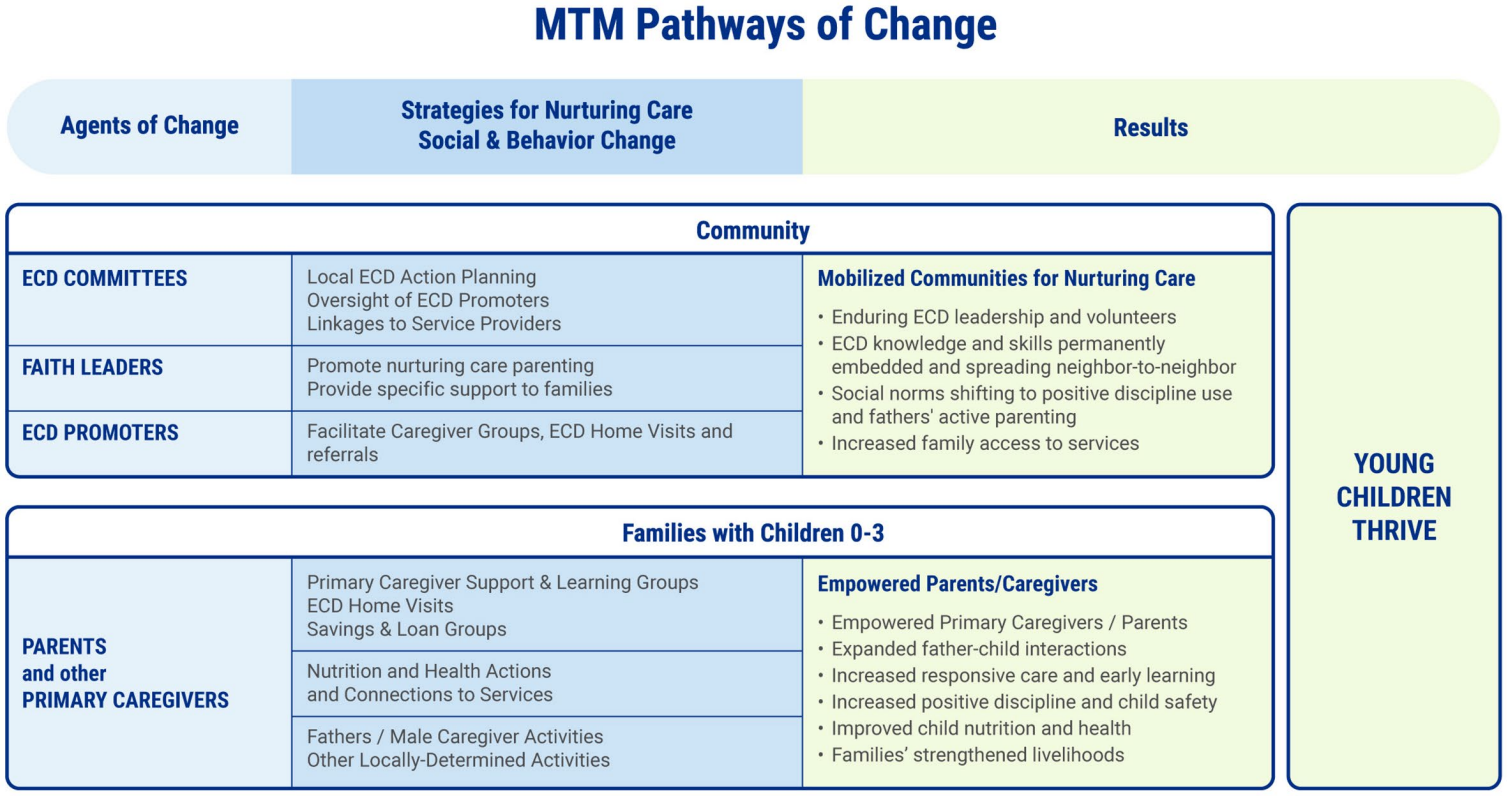
*Moments That Matter^®^* (MTM) logic model.

*Moments That Matter^®^* combines standard core functional elements and quality standards with flexibility to apply them in context-specific ways in different country and local rural settings. MTM equips community agents of change as well as empowers Primary Caregivers to be self-directed agents of their own parenting change. MTM’s theory of change and results are summarized in Figure 1.

*Moments That Matter^®^* is a civil society, community-implemented program that starts from a faith base. The program leverages these faith-based assets to mobilize leaders and communities, which are inclusive of all faiths, denominations and those without religious affiliation in a project area.

Local ECD Committees are formed and trained with three main functions:

- To provide oversight and support to ECD promoters.
- To collaborate with MTM-trained and other faith leaders.
- To coordinate with ECD-related service providers and stakeholders.

In Zambia, the ECD Committees are multi-sector, with representatives from different parts of local civil society and government. Each ECD Committee has at least one faith leader member representing the other MTM-trained faith leaders. After each Committee meeting, they share with other faith leaders the resolutions and emerging issues, while follow up actions they need to take. In Kenya, the equivalent ECD Committees for MTM purposes are called *ECD Faith Leader Consortia,* a group formed solely by MTM-trained faith leaders. Though, there are already existing government-organized multi-sector early childhood development groups, which are focused more on pre-primary education. Once local ECD Committees are established, volunteers are identified and both volunteers and faith leaders are trained as ECD Promoters.

## 3. Key monitoring, evaluation, and learning elements

The MTM community-led MEL process is carried out by three types of MTM stakeholders, identified below with the number active in 2022:

- *Community Leadership*: ECD Committees [Kenya—5; Zambia—87] and MTM-trained faith leaders [Kenya—160; Zambia—175].
- *Frontline Implementers*: ECD Promoters, including ECD Lead Promoters (who have some additional supervisory and reporting responsibilities) [Kenya—223; Zambia—1,361].
- *Focal Participants*: Primary Caregivers [Kenya—2,899; Zambia—10,895] with children under three and their families.

**Figure 2:**
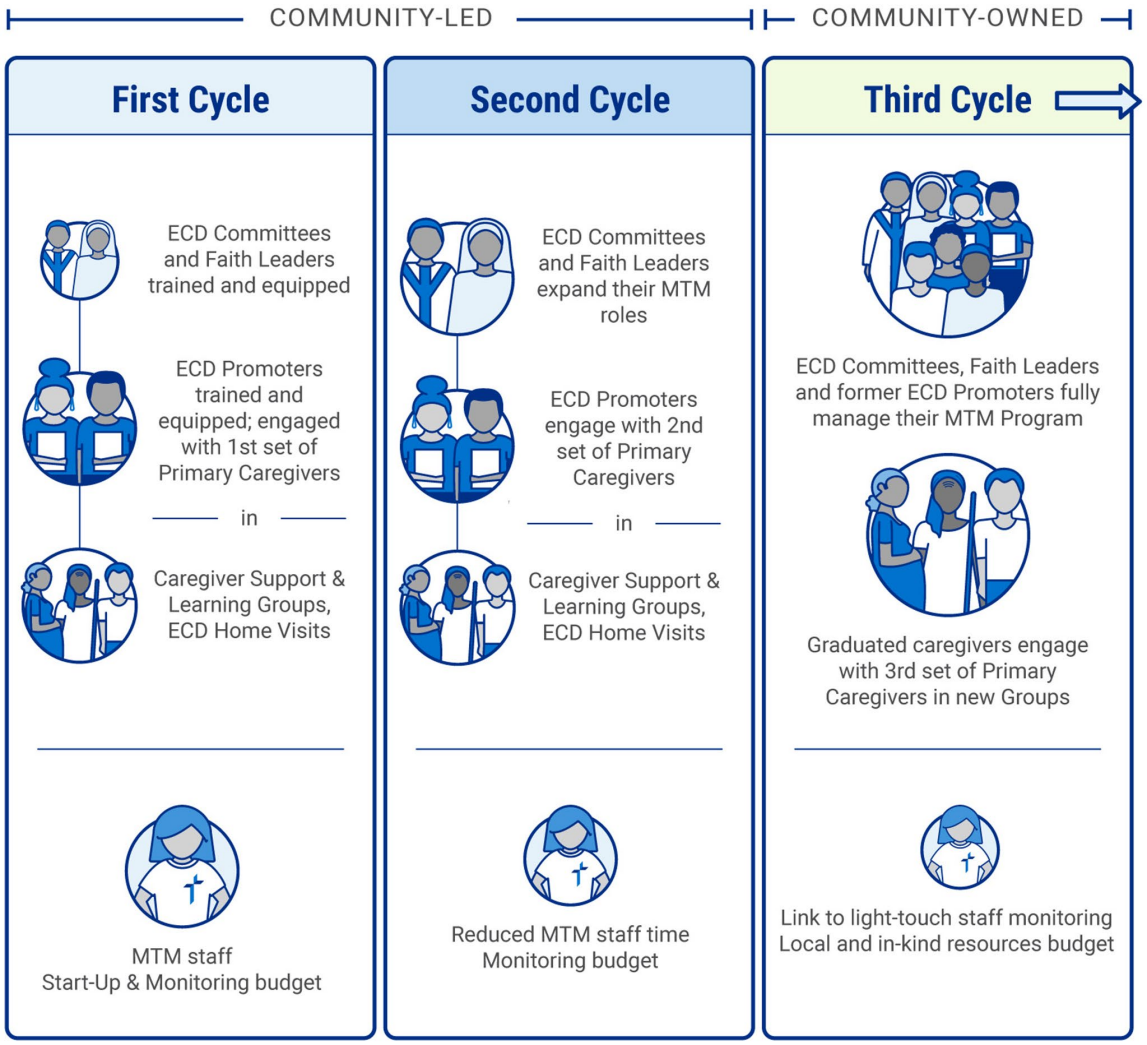
*Moments That Matter^®^* (MTM) implementation and monitoring cycles.

Early childhood development Committees and MTM-trained faith leaders promote nurturing care parenting and social and behavior change with both MTM registered families as well as other families. As frontline implementers, ECD Promoters serve as a bridge between the MTM Primary Caregivers and MTM community leadership and program staff. MTM is implemented and monitored in cycles. ECD Committees become a joint implementing body with MTM Program staff during the first two program cycles, then transition to an independent implementer starting in the third cycle. Each cycle engages a new set of vulnerable Primary Caregivers with children ages zero to three in the MTM community. As seen in Figure 2, over the first two cycles, a MTM community takes on greater responsibility with diminishing program staff and budget support. In the third cycle and beyond, MTM is community-owned.

The community-led MEL process is people-centered and functions in tandem with a more traditional, staff-executed project MEL system during the first two cycles. Initially, MTM Program and MEL staff facilitate a process whereby the ECD Committees, MTM faith leaders and Lead Promoters design and plan their community MEL framework. Staff provide capacity-building, mentoring in organizational management, supportive supervision and MEL to ensure successful uptake and eventual transition to community-ownership. The two types of MEL overlap, serving to meet multiple purposes:

- *Staff* track standard MTM indicators to meet organization needs for effective project management and improvement, plus any additional funder-required data.
- *Communities* track and use some of the standard indicators, as well as setting and monitoring their own specific goals with indicators of success meaningful to them.

Indicators for measuring MTM results are mostly parenting practices (rather than knowledge or attitudes) since it is these behaviors which generate the impact on children’s development. A few examples are provided in Table 1; “MTM Community Leadership” refers to ECD Committee members, MTM-trained faith leaders and ECD Promoters–or some combination depending on the particular situation.

**Table 1:** Examples of indicators with users of the data.

Standard program indicators	Data collector	Data used by
Number of groups sensitized to nurturing care, with topics discussed	ECD committees	MTM community leadershipMTM staff
Resolution of critical issues arising with MTM families or program implementation		
Primary Caregiver tried the parenting practice introduced at previous meeting	Primary caregiversECD promoters	Primary caregiversECD promoters
Health, nutrition and other referrals made for children and caregivers	ECD promoters	MTM community leadershipMTM staff
*MTM community-set indicators*		
Child cries when father-secondary caregiver leaves him/her (*demonstrates bonding*)	Primary caregiversFather-secondary caregiverECD promoters	Primary caregiversFather-secondary caregiversECD promoters
Increased playtime with my child	Primary caregiversECD promoters	Primary caregiversECD promotersMTM community leadership
Tried positive discipline methods		
Received assistance from my spouse to care for the children		

**Figure 3:**
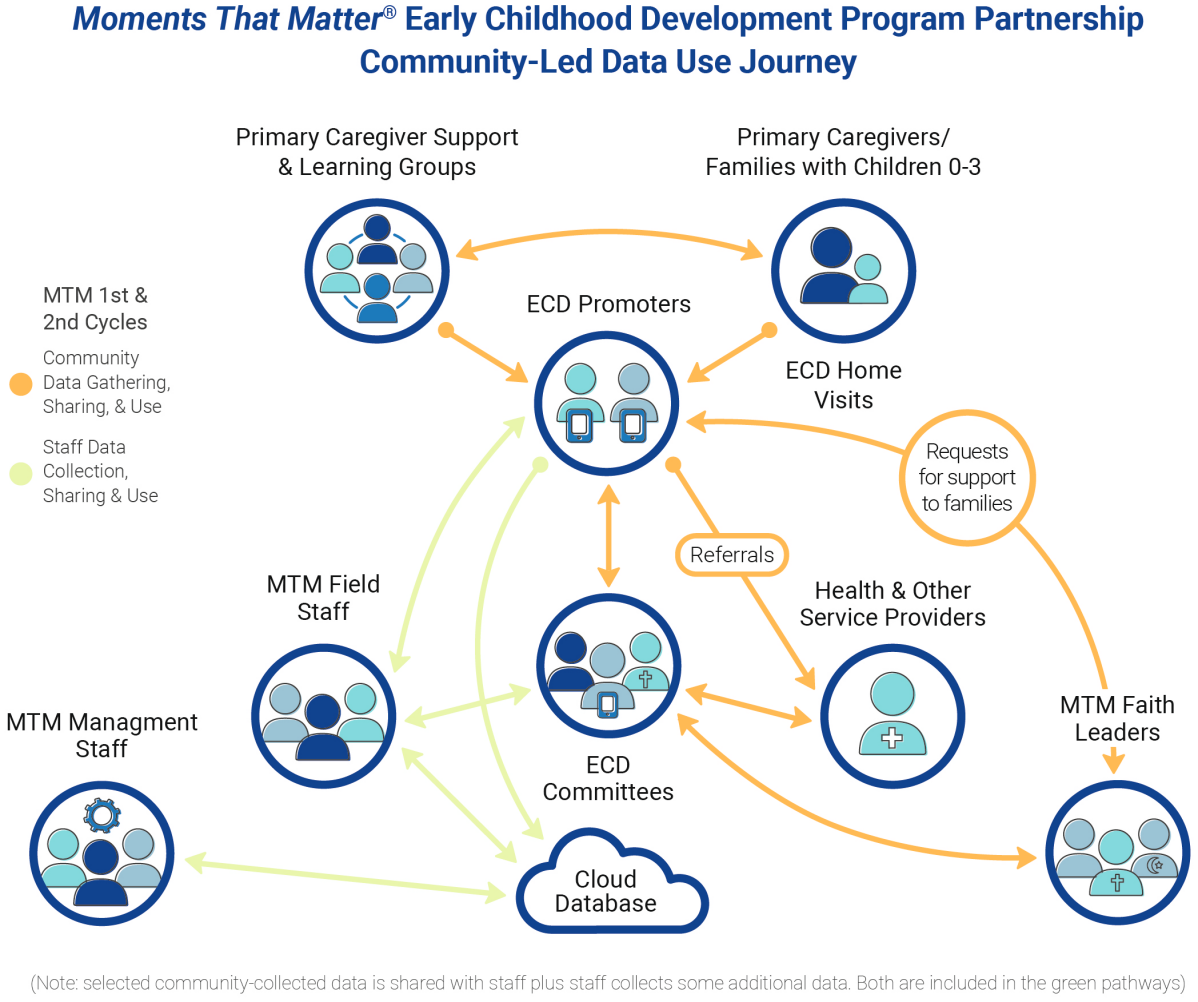
*Moments That Matter^®^* (MTM) community-led data use journey.

The data is primarily collected by the MTM community leadership, shared and used within the community MEL process for local action, as depicted in Figure 3, with ECD Committees taking the lead. Selected community-collected data is also aggregated and reported via the staff project MEL system. Examples of this intersection include:

- ECD Committees monitor project activities on a monthly basis. From the monitoring visits, ECD Committees share findings, recommendations and learnings with both ECD Promoters and program staff.
- Lead ECD Promoters regularly review and consolidate data collected from the ECD Promoters before submitting to project field staff and program managers.

The monitoring tools ensure fidelity to implementing the interventions as designed and for quality assurance based on MTM quality standards. Using the home visit and group facilitation checklist observation versions, for example, program staff and Lead Promoters reinforce ECD Promoters’ positive actions and help them improve in weaker areas through additional coaching and problem-solving. Starting in 2023, Primary Caregivers themselves will use the *Actions to Practice Passport* tool at their Group meetings and their ECD home visits. The communities currently use paper forms with the exception of Lead Promoters who have tablets for mobile data collection; project staff also use mobile data collection. See Table 2 for types of data collected, by whom and with what tool.

**Table 2:** Interconnected staff and community monitoring data collection.

Type of output data	Responsible	Data collection tool
ECD activities in communities, linkages to service providers	ECD committees	Committee and project records outreach tracking tool
ECD promoter reflection/in-service meetings	Project field staff, lead promoters	Reflection meeting guide
Primary caregiver support and learning groups	ECD promoters	Group meeting progress tool, group facilitation quality checklist
	Lead promoters, project field staff	Group facilitation quality checklist [*observation tool version*]
	Primary caregivers	Actions to practice passport
ECD home visits	ECD promoters	Home visit progress tool, Home visit quality checklist
	Lead promoters, Project field staff	Home visit quality checklist *[observation tool version]*
	Primary caregivers	Actions to practice passport
Referrals for children, caregivers	ECD promoters	Home visit progress tool
Project activity outputs	Project field staff managers	MTM output data collection forms

## 4. Measurement for change and community MEL

In MTM, community committees, frontline implementers and focal participants all monitor for change from the outset of the program. This combined effort strengthens impact and fuels the transition to community ownership. All community participants engage in community MEL activities using specific MEL tools, but their MEL engagement shifts throughout the life cycle of the program. At present, longer term community ownership of MTM and its MEL is being demonstrated in more than 70 MTM communities in Zambia, the oldest MTM program country. In Kenya, the first set of MTM communities will enter its third cycle in April 2023.

## 5. Strengthening impact and transition to ownership through monitoring for change

### 5.1. Early childhood development committees leading community MEL

At the outset, ECD Committees set up their basic MEL when they first form and launch the program in their community in the first implementation cycle. In the first cycle, MTM Program staff work closely with the ECD Committees at their monthly meetings to ensure Committees successfully take up their responsibilities and fulfill their functions. During the second cycle, staff gradually reduce their direct involvement while gather a core set of indicator data.

#### 5.1.1. First and second cycles’ community-led MEL

During the first two cycles, Committees use the standard MTM community MEL tools to set and carry out specific goals with annual action plans for their communities, track progress and prepare reports used by them as well as the staff. Within this standard framework, each ECD Committee tailors the MEL process, data gathering, assessment, learning and project improvements for what will work best in their context. On a monthly basis, ECD Committees also collate MEL data, discuss achievements of their targets, progress in uptake of nurturing care and plan how to address challenges. They document learning as well as the adaptive actions. Fundamentally, ECD Committee members see direct results from their committee work, which is not dependent on program staff.

In Mapulanga, Zambia, for example, the ECD Committee adapted the outreach tracking tool, with the assistance of the program manager, to better monitor MTM progress in their community and take action as problems arose. Through the utilization of the tool and interaction with ECD Promoters and caregivers, the ECD Committee linked 12 children with severe malnourishment to the Scale Up Nutrition Program at health facilities. Their monitoring for change approach identified 40 MTM families suffering from extreme poverty due to pandemic effects, and the ECD Committee was also able to connect Primary Caregivers to the government social cash transfer program.

#### 5.1.2. Second cycle community-owned MEL

During the second cycle, staff gradually reduce their direct involvement while gather a core set of indicator data, and the activities continue much as before—deepening practice and ownership.

#### 5.1.3. Third cycle community-owned MEL

In the third cycle, now reached in Zambia, MTM communities carry on independently with their own program implementation and MEL, with only periodic sharing of progress with program staff. In Zambia, the project team check in quarterly with ECD Committees and MTM faith leaders rather than monthly. It is the ECD Committees that provide brief monitoring reports with a limited set of key data using third cycle tools. ECD Committees also continue to manage and oversee all MTM ECD activities in their respective communities. A set of graduated Primary Caregivers are equipped to facilitate caregiver groups composed of vulnerable Primary Caregivers and their children under three. The ECD Committees continue to make use of the participatory tools and resources acquired during the implementation and project funding phases. For instance, they use Venn diagrams in identifying and selecting new caregivers, stakeholder analysis exercises, self-reporting templates and registration books for basic income and expense tracking.

Furthermore, ECD Committees maintain the existing relationships with various stakeholders by using a management/MEL tool that they adjust to align with their particular context. They manage communication plans and feedback loops which allow them to obtain and share information on challenges, opportunities and lessons learned. In 2023, drawing on the Zambia experiences and MEL tools, Episcopal Relief & Development is working with the ECD Committees themselves to develop a community monitoring system that supports community-owned MEL in their third cycles and beyond.

### 5.2. *Moments That Matter^®^*-trained faith leaders’ monitoring for change

#### 5.2.1. First and second cycles’ community-led MEL

At the outset, local faith leaders are engaged as key influencers in their communities with ongoing connections to families. MTM mobilizes clergy and lay leaders, both women and men, as agents of change for nurturing care parenting and early childhood development. Faith leaders provide counseling and other direct support, as well as sermon guides and scripture studies, to promote nurturing care parenting particularly focused on harsh punishment/positive discipline and expanding the fathers’ role with young children.

Faith leaders document issues that arise, as they promote nurturing care, and meet with families in the course of their pastoral work. They also, identify gaps and bring those to the ECD Committee meetings for action planning. For example, a male faith leader was able to persuade a father that was resistant to attending a caregiver group session, following up on a concern arising at the ECD Committee. In another case, a faith leader supported a father that had rejected a baby born with spina bifida. The pastor counseled him and helped refer the family to services, again working with the ECD Committee. Both faith leaders’ direct work with MTM families and their nurturing care social and behavior change communication with their faith groups are monitored through ECD Committees.

#### 5.2.2. Third cycle community-owned MEL

By the third cycle, MTM faith leaders synthesize their own MTM work quarterly using a tool to document specific activities, as well as new issues, successes, challenges and recommendations for program implementation improvement. These are shared at ECD Committee meetings. The engagement with faith leaders and faith-based social and behavior processes also helps to broadly spread ECD knowledge and MTM social and behavior change communication in a more informal, economical way through faith networks.

### 5.3. Early childhood development promoters leading interactive and dynamic monitoring for change

#### 5.3.1. First and second cycles’ community-led MEL

Early childhood development Promoters are frontline implementers in the first two MTM cycles in their community. They carry out project MEL while serve as leaders in the community-MEL, fostering caregivers’ participatory MEL. ECD Promoters commit to serving one implementation cycle with a set of Primary Caregivers. The vast majority of them continue to serve through a second cycle with a new set of Primary Caregivers. ECD Promoters build their relationship with Primary Caregivers by facilitating learning, practicing parenting skills and solving their own parenting problems, while making referrals as needed. ECD Promoters use standard monitoring tools that focus on behavior change. They focus monitoring practices on the high-impact parenting actions learned and practiced by MTM caregivers.

ECD Promoters convene at monthly reflection meetings to assess and use data gathered from tracking tools, which guide improvements in their work with families. Drawing on the tracking tools and their own memories, ECD Promoters share challenges they have encountered with their caregivers/families, generate solutions and support one another with resources or referrals. They share successes observed in caregivers and best practices in their facilitating role. Lead Promoters and project field officers document key points and agreed action steps, which are later followed up to improve overall programming.

During the first cycle, caregivers commit to trying new parenting practices at home that they learn about each week in their caregiver group support and learning group session led by ECD Promoters. Progress and challenges with the curriculum and commitments are documented and followed by ECD Promoters and caregivers themselves during ECD home visits.

Progress, challenges and actions are captured in the following tracking tools:

- *Caregiver support and learning group meeting progress tracking tool:* Captures attendance, topics covered and challenges to be addressed. ECD Promoters facilitate a learning-practice-reflection loop with eight to thirteen Primary Caregivers during these meetings using the progress tracking tool. Progress through the curriculum and caregiver reflections is tracked on each unit.
- *Early childhood development home visit progress tracking tool:* In the home, ECD Promoters observe the relationship between Primary Caregiver and child or children directly, assessing their progress towards more nurturing care parenting, learning in more detail about caregivers’ questions and concerns and responding accordingly. The home visit provides an opportunity to dialog about specific barriers and how to overcome them, respond to specific concerns and make referrals as needed. The tracking tool captures this key information for follow up. For instance, if a referral was made in the previous visit, the tracking tool reminds the ECD Promoter to check and see if a Primary Caregiver took the child to receive the service needed.

#### 5.3.2. Third cycle community-owned MEL

At the end of a community’s second cycle, the ECD Promoters’ direct volunteer role ends. They pass on to selected Primary Caregivers the basics of how to facilitate new caregiver groups formed by a new set of most vulnerable Primary Caregivers with children under three. These new groups will now be peer-run by graduated caregivers. The former ECD Promoters then serve as resources to be called on as needed by caregiver facilitators and they also often serve on ECD Committees to contribute to the community-owned programming. As residents of the MTM communities, former ECD Promoters informally check on the peer caregiver-led groups every 2 months to ensure that they are going well and to provide mentorship support or additional information required by the caregiver groups.

### 5.4. Community MEL lessons learned

MTM partners expected there to be some variations between the two country programs (though the rural MTM communities share many sociodemographic similarities). However, the positive experiences with leading their own MEL and their challenges described below were common to both settings. The exception are lessons on the third cycle transition—due only to the fact that the Kenyan MTM program is newer and has not yet reached that stage.

- In addition to ECD Committees, MTM faith leaders and ECD Promoters are critical for the facilitation of community-led MEL. Their initial training should include key elements of community-led MEL so that its practice can be strengthened over the first two cycles prior to ownership.
- Early childhood development Committees need specific skills training in data analysis, interpretation and reporting of MTM standard and project-specific indicators. This training should be conducted early in the program to allow time for mentorship and capacity-building throughout the program life cycle.
- Community-led MEL reporting formats should harmonize with relevant government reporting templates to ensure appropriate ECD data and information is incorporated into government health information systems.

Lessons specifically on the transition to an independent third cycle in Zambia:

- Due to low literacy levels among caregivers in MTM project communities, peer facilitator caregivers need simplified reporting tools. For this reason, caregivers were first paired with lead ECD Promoters that mentored and provided guidance. Simplified reporting tools are being created as part of the 2023 monitoring system development.
- Exchange visits across ECD Committees from different MTM communities provide valuable knowledge transfer, skills building and inspiration. These visits contribute to the strengthening of leadership and organizational management and ECD advocacy work, engaging government health and other service providers and local civic organizations.

## 6. Primary caregivers’ self-monitoring for change

At the outset and periodically thereafter, Primary Caregivers set their own individual parenting goals and share progress. In Kenya, during caregiver learning and support group meetings, caregivers set goals on how to improve their parenting practices. The caregivers share their situation and compare notes on what works and what does not work. In Zambia, ECD Promoters ask caregivers to share what they would like to achieve in that particular quarter concerning parenting. In both country programs, caregivers would share and celebrate achievements and also set higher targets for continuous improvement.

In addition to the examples in Table 1, other specific goals caregivers have set include:

- Reduce the use of physical punishment.
- Create a safe environment for child nurturing, such as clearing bushes, leveling ground, removing stones and sharp objects for a safer place for children to play.
- Play with the child for 1 h in the morning and 2 h in the afternoon.
- Develop “co-parenting” goals of what the mother and father will be doing, on which day of the week.

Another type of monitoring for change is found in family “research experiments” on nurturing care practices. When caregivers observe positive changes in their children and relationships, they are motivated to continue. For example, fathers are normally requested to create time to play with children but often find it difficult. However, if they can be convinced to try, after a few “trial” playtimes with their children, fathers develop a passion for it and intentionally create regular time to play with children.

Each Primary Caregiver Support and Learning Group elects its own officers to lead the group together with the ECD Promoter. Leading up to graduation from the ECD Promoter-led phase (completing the 24-month or 18-month curriculum), members in each group decide whether they want to continue as a fully member-run group. Regardless, when their MTM community reaches the third cycle, there is a locally-set selection process for interested graduated Primary Caregivers to become peer leaders for new groups, which are formed with other vulnerable Primary Caregivers of children under three. These peer-led new Caregiver Support and Learning Groups are monitored by ECD Committees. In instances where peer mother-led groups face challenges, the caregivers consult with the former Lead or ECD Promoter within their communities.

## 7. *Moments That Matter^®^* community-led MEL and successful scaling up

The community-led MEL contributes to successful scaling through two channels.

### 7.1. *Moments That Matter^®^* scaling to new communities

As current MTM communities graduate to community ownership and implementation, project staff and budget are invested in new geographic areas, working with new communities to start their own MTM programs. In conjunction with the Ministry of Health, marginalized, underserved areas are selected for MTM based on a set of criteria including: poverty level, poor child health and nutrition indicators, HIV prevalence, lack of other ECD programming and access, and community leadership interest in and commitment to carrying out the MTM Program.

### 7.2. *Moments That Matter^®^* scaling through deepening reach within MTM communities

The community-led MEL system contributes to the sustainable development of community ownership of their local MTM programming. This means engaging new vulnerable families or Primary Caregivers during pregnancy and/or with children under three in the same area primarily through volunteers implementing more MTM Program cycles–without project staff and budget resources from faith-based organizations leading the MTM Program Partnership in the country.

*Moments That Matter^®^* Zambia began its third cycle in 2017. To date, 73 communities have completed the transition to third cycles; of these, 81% of the ECD Committees have registered as community-based organizations (CBOs) with another 12 communities in the process. As CBOs, they will be able to manage their MTM programs more effectively and raise funds to sustain them.

ZACOP has done some participatory assessments of third-cycle MTM communities with the aim of sharing ideas, motivating caregiver groups, collecting information on the changes sustained and gathering any learnings and adaptations made by peer-led groups. This information is then shared with other groups and MTM communities to improve the implementation of third-cycle activities.

In Kenya, the MTM Program is planning for its first set of MTM communities to transition to their third cycle beginning in April 2023. Drawing on the Zambian MTM experiences, the Kenyan MTM program staff and ECD Committees are undertaking the following in preparation:

- Organizing before action review meetings that reflect on successes, challenges and adaptive actions.
- Orientating on third cycle project design, key indicators as well as reporting, sharing and agreeing on community MEL tools.
- Planning for periodic reflection meetings.

## 8. Discussion

Episcopal Relief & Development and its partners concluded that MTM community MEL demonstrated to varying extents the measurement for change aspirations. The M4C approach—people-centered, inclusive, interactive, informative and dynamic has been critical to achieving a quality, well-functioning community MEL system during the first two cycles with program staff. The MTM experience in Zambia illustrates that the community MEL system also undergirds and fuels a dynamic process of transformation from the initial community-led, time-bound project with staff and budget to third cycle community ownership of independent MTM programming. Thus, it also supports with MTM scale up through the two channels of expanding reach within MTM communities and launching MTM in new areas.

*Moments That Matter^®^* is designed with the *people-centered* dimension. Community leaders, ECD service providers, volunteers and Primary Caregivers all set their specific goals and priorities to tailor the MTM program to their distinct context, while using user-friendly tracking of progress. In this way, the community MEL is *inclusive*, with all stakeholders engaged in defining success at their level, monitoring and making adjustments over time. The *interactive* nature is demonstrated by the relationship-based social and behavior change strategies such as Primary Caregivers with each other and their ECD Promoters; faith leaders with MTM families; and community leaders with ECD service providers. The recurring, regular monthly or quarterly check-ins at three levels–caregiver/family, ECD Promoters, ECD Committees–provide the *informative* dimension, ensuring the capacity for continuous gathering and use of key information from different sources to guide decision-making and solve problems.

Through the APRHC implementation research study and MTM’s own MEL community case study, MTM partners also concluded that further strengthening of measurement for change was needed. Contribute to MTM’s increased impact and sustainability while scaling. Recommendations for improving the MTM intervention as it continues to scale are reflected in the following actions.

In the MTM implementation phase now underway in 2023, program staff and MTM communities are working to deepen the *dynamic* dimension in particular. For the ECD home visits, MTM is providing a more user-friendly tool for the ECD Promoter and introducing the Actions to Practice Passport, a new visually-oriented tool for Primary Caregivers to track and share their progress using nurturing care parenting practices and to achieve their goals. These tools will support the caregivers and ECD Promoters’ capacities to be more responsive to challenges and opportunities in the process of strengthening nurturing care parenting.

In 2023, MTM will share new and updated social and behavior change resources that are more effective and user-friendly, incorporating some of the lessons learned and ultimately enhancing the dynamic dimension. These include structured ECD Committee training and tools with integrated program and MEL management, such as the updated *ECD Promoter Home Visit Booklet* and the new Primary Caregiver *Actions to Practice Passport* tool. The Passport tool provides a visual, an easy way to record what was previously only a verbal commitment at group meetings, to practice what was learned at the session and report back on it later. The ECD Committee tool has an expanded section to track referrals and strengthen referral pathways. Other improvements to strengthen the interactive and inclusive dimensions, such as expanded community MEL training, will be undertaken in 2023–2024 as part of planned further development of the third cycle MEL framework and the community monitoring system.

The community-led MEL system is founded on the shared community vision of children achieving their potential, fueled by a positive, self-reinforcing dynamic of goal setting, progress-tracking, learning, problem-solving, adaptation and celebration. This system contributes to the sustainability of program outcomes at the child, parent/caregiver, family and community levels. The sustained program outcomes are due to the ownership of changes, residing not only in MTM leaders and volunteers but also with the participating Primary Caregivers and families themselves. The *Moments That Matter*^®^ community-led program, with its monitoring and measurement for change, drives both sustained impact and effective scaling of the MTM Early Childhood Development Program Partnership.

With its permanent presence and commitment to serve the most vulnerable, the extensive faith network in rural Africa provides a systematic pathway for MTM scaling in civil society. These MEL processes have contributed to successful scaling with quality through two channels: deepening reach to new families through sustainable community-owned MTM and investing in MTM community start-up in new geographic areas.

With so many children at risk in sub-Saharan Africa, there is a tremendous need for ECD parenting empowerment and community ownership of nurturing care. MTM partners recommend that other ECD programs incorporate community MEL from the outset of implementation and invest in local capacity strengthening. As demonstrated by the MTM Program experiences, community-led measurement for change processes reinforce ownership and motivation mutually within families and ECD community leadership. This results in channeling these forces into a powerful catalyst for and commitment to sustained nurturing care so that young children thrive.

## Data availability statement

The original contributions presented in the study are included in the article/supplementary material, further inquiries can be directed to the corresponding author.

## Author contributions

All authors listed have made a substantial, direct, and intellectual contribution to the work and approved it for publication.

## Funding

The funds for the open access publication fees were provided by Episcopal Relief and Development, an international nongovernmental organization.

## Conflict of interest

The authors declare that the research was conducted in the absence of any commercial or financial relationships that could be construed as a potential conflict of interest.

## Publisher’s note

All claims expressed in this article are solely those of the authors and do not necessarily represent those of their affiliated organizations, or those of the publisher, the editors and the reviewers. Any product that may be evaluated in this article, or claim that may be made by its manufacturer, is not guaranteed or endorsed by the publisher.

## Footnotes

^1^The person in the home who has primary responsibility for, cares for, and spends the most time with the child (children) on a daily basis. The Primary Caregiver is not always the mother, it could be a father, grandparent, other relative, sibling or unrelated adult.
